# Temporal Orchestration of Krüppel-like Factors During Cardiac Remodeling Following Isoproterenol-Induced Myocardial Injury

**DOI:** 10.3390/genes17060657

**Published:** 2026-06-03

**Authors:** Michelle G. Santoyo-Suárez, Juan Andrés García-Loredo, Jimena Deyanira Mares-Montemayor, Juan Luis Delgado-Gallegos, Lourdes Garza-Ocañas, Oscar Rodríguez-Nuñez, Adolfo Soto-Dominguez, Alberto Camacho-Morales, Patricio Zapata-Morin, Gerardo R. Padilla-Rivas, Elsa N. Garza-Treviño, Jose Francisco Islas

**Affiliations:** 1 Biochemistry and Molecular Medicine Department, School of Medicine, Autonomous University of Nuevo Leon, Monterrey 64460, Mexicoalberto.camachomr@uanl.edu.mx (A.C.-M.);; 2 Laboratory of Mycology and Phytopathology, School of Biological Sciences, Autonomous University of Nuevo Leon, San Nicolas de los Garza 66451, Mexico; 3 Pharmacology and Toxicology Department, School of Medicine, Autonomous University of Nuevo Leon, Monterrey 64460, Mexico; 4 Histology Department, School of Medicine, Autonomous University of Nuevo Leon, Monterrey 64460, Mexico

**Keywords:** Krüppel-like factors, myocardial damage, isoproterenol damage, cardiovascular diseases, hypertrophy

## Abstract

**Background**: Myocardial infarction triggers a complex remodeling process involving inflammation, hypertrophy, fibrosis, and electrical adaptation, ultimately predisposing the heart to failure. Krüppel-like factors (KLFs) are transcriptional regulators implicated in cardiovascular development and disease; however, a comprehensive temporal characterization of their coordinated activity during post-injury remodeling remains lacking. **Objective**: To define the temporal orchestration of the KLF family during myocardial injury and hypertrophy, and to integrate these dynamics within regulatory networks associated with cardiac remodeling. **Methods**: Myocardial injury was induced in rats using intraperitoneal isoproterenol. Left ventricular tissue was collected over a 21-day period. Cardiac morphometry, histology, immunohistochemistry, and quantitative gene expression analyses were performed to evaluate structural and transcriptional changes. Publicly available human cardiac and fibroblast datasets were analyzed for translational comparison, and protein–protein interaction networks were constructed to identify functional associations. **Results**: Isoproterenol treatment induced progressive hypertrophy, structural disorganization, and sustained fibrotic remodeling. KLFs displayed coordinated, phase-specific regulation, characterized by early activation of inflammation-associated members, intermediate engagement of factors linked to transforming growth factor signaling and hypertrophy modulation, and late induction of regulators associated with apoptosis and scar formation. These temporal patterns paralleled changes in inflammatory mediators, cardiac transcription factors, and genes involved in electrical and calcium handling pathways. Human expression analyses supported tissue-specific specialization of key KLFs. **Conclusions**: KLFs exhibit a coordinated and temporally structured regulatory program during myocardial remodeling, functioning as a transcriptional network that integrates inflammation, fibrosis, hypertrophy, and electrical adaptation. These findings position KLFs as key regulatory nodes in cardiac remodeling and potential targets for therapeutic intervention.

## 1. Introduction

Cardiovascular diseases remain the leading cause of global mortality, with ischemic heart disease, stroke, and heart failure accounting for a disproportionate burden of morbidity and mortality [1,2,3]. Among them, ischemic heart disease is specifically significant due to its acute presentation and severity, particularly in the context of acute myocardial infarction (MI), claiming over a million lives each year in the United States alone [3]. MI results from the abrupt interruption of coronary blood flow, leading to oxygen deprivation, cardiomyocyte death, and irreversible myocardial injury if not promptly treated.

The subsequent reparative and compensatory responses encompass a coordinated cascade of inflammation, extracellular matrix deposition, and cardiomyocyte hypertrophy. Although the initial processes are directed toward preserving cardiac function, they ultimately drive maladaptive remodeling and progression to heart failure [4]. Elucidating the molecular regulators that govern this transition remains a central challenge in cardiovascular biology.

Krüppel-like factors (KLFs) comprise a family of zinc-finger transcription factors that regulate diverse biological processes, including cellular growth, differentiation, apoptosis, and tissue homeostasis [5,6]. Structurally, KLFs are characterized by three conserved Cys2His2 zinc-finger domains that bind GC-rich DNA elements such as CACCC-, GC-, and GT-box sequences [7]. Since their identification in 1993, eighteen KLF family members have been described, each exhibiting distinct expression patterns and transcriptional regulatory functions [5,8].

Increasing evidence supports a central role for KLFs in cardiac hypertrophy and remodeling, where their activity is highly context-dependent and mediated through interactions with multiple signaling pathways. For instance, KLF2 functions as a negative regulator of cardiac hypertrophy and is predominantly expressed in endothelial cells [9,10]. Mechanistically, KLF2 modulates transforming growth factor-β (TGF-β) signaling by promoting SMAD7 expression and inhibiting activator protein-1 (AP-1) activity [10,11]. In contrast, KLF5 is associated with pathological remodeling and is upregulated in conditions such as end-stage heart failure and diabetic cardiomyopathy [12,13]. Experimental models have demonstrated that reduced Klf5 expression attenuates angiotensin II–induced cardiomyocyte growth and fibrosis, highlighting its role as a mediator of hypertrophic signaling [13,14].

At the molecular level, KLF5 regulates the transcription of several genes involved in cardiovascular remodeling, including platelet-derived growth factor (PDGF)-A/B, early growth response-1 (Egr-1), plasminogen activator/inhibitor-1 (PAI-1), inducible nitric oxide synthase (iNOS), and vascular endothelial growth factor (VEGF) receptors [15]. These observations underscore the diverse and context-dependent roles of KLF family members in coordinating structural and functional adaptations of the heart.

Given the complexity of myocardial injury, a family-wide profiling of KLF expression provides a unique opportunity to capture both well-established regulators and lesser-characterized members that may contribute to disease progression. In this study, we examined the gene expression dynamics of the entire KLF family following myocardial damage and hypertrophy in female rats, aiming to identify the coordinated regulatory networks that modulate inflammation, conduction, and structural remodeling. To further highlight the potential translational relevance of our findings, we also compared KLF expression patterns in rats with publicly available human datasets (GTEx), providing a framework to assess cross-species conservation of these transcriptional programs.

## 2. Materials and Methods

### 2.1. Animals and Experimental Design

Female Wistar rats (150 ± 20 g) were housed at the Pharmacology Animal Care Unit, Facultad de Medicina, Universidad Autónoma de Nuevo León. Animals were maintained under controlled environmental conditions (20–24 °C, 12 h light/dark cycle) with ad libitum access to food and water. A 7-day acclimatization period was allowed prior to the beginning of the experiments.

Myocardial injury was induced by a single intraperitoneal injection of isoproterenol hydrochloride (65 mg/kg; Sigma-Aldrich, St. Louis, MO, USA) dissolved in physiological saline. Control animals received an equivalent volume of saline. Animals were monitored daily for clinical signs of cardiac stress throughout the study.

The study was designed as a longitudinal time-course model of cardiac remodeling over 21 days. Although all animals were followed until day 21, tissue collection was performed at predefined time points to capture distinct phases of remodeling: early inflammatory, proliferative, and maturation phases. Sampling centered on the early inflammatory to mid proliferative stage (days 1–9), while the rest of the proliferative to maturation phase was sampled every 2 to 3 days. Each group consisted of n = 3 biological replicates and 2 technical replicates.

All procedures were approved by the Institutional Review Board of the Facultad de Medicina, Universidad Autónoma de Nuevo León (protocol BI21-00006, approved 21 May 2021, with annual renewals) and conducted in accordance with the National Research Council Guide for the Care and Use of Laboratory Animals.

### 2.2. Tissue Collection and Processing

Animals were anesthetized and euthanized via cervical dislocation at predefined time points. Hearts were then excised via thoracotomy, and great vessels were ligated and transected to preserve tissue integrity.

Excised hearts were rinsed in ice-cold saline, blotted dry, and weighed. Cardiac dimensions (length, width, and height) were recorded using a digital caliper. Left ventricular tissue was dissected and divided for downstream analysis.

A portion of each sample was fixed in 10% neutral-buffered formalin for histological (H&E) analysis. The remaining tissue was snap-frozen in liquid nitrogen and stored at −80 °C for RNA extraction.

### 2.3. Cardiac Morphometry

Cardiac hypertrophy was assessed by calculating the heart volume using the ellipsoid formula:

V = (3/4)π × D1 × D2 × L

where L represents length, and D1 and D2 correspond to width and height, respectively. The heart volume was normalized to body weight (CV/BW) to account for inter-animal variability.

### 2.4. RNA Isolation and Quantitative RT-qPCR

The total RNA was extracted from left ventricular tissue using TRIzol^®^ (Invitrogen; Thermo Fisher Scientific, Waltham, MA, USA) following the manufacturer’s protocol. RNA concentration and purity were assessed spectrophotometrically; 250 ng of the total RNA was reverse transcribed into cDNA using the SuperScript™ VILO™ cDNA Synthesis Kit (Thermo Fisher Scientific, Waltham, MA, USA).

Quantitative real-time PCR (RT-qPCR) was performed using the Applied Biosystems 7500 Fast Real-Time PCR System (Thermo Fisher Scientific, Waltham, MA, USA) under SYBR Green conditions. Cycling parameters were: 95 °C for 20 s, followed by 40 cycles of 95 °C for 3 s and 60 °C for 30 s.

Gene expression levels were normalized to GAPDH and calculated using the 2^−ΔΔCt^ method. For each experimental time point, analyses were performed using 3 biological replicates. From each animal, 2 technical replicates were analyzed from distinct left ventricular tissue sections.

Target genes included members of the KLF family (Klf2, 3, 4, 5, 6, 9, 11, 12, 13, 15), hypertrophy markers (ANP, α-MHC), inflammatory mediators (Il-1β, Il-6, Tnf-α, Nf-κB), and conduction-related genes (Cx43, Slc8a1, Tbx5). Primer sequences are listed in Table S2.

### 2.5. GTEx Analysis

Human gene expression data were obtained from the Genotype-Tissue Expression (GTEx V8) project using the Multi-Gene Query tool available at https://www.gtexportal.org/home/. Expression values were obtained as TPM (transcripts per million) as provided by the GTEx portal without additional normalization beyond the GTEx processing pipeline. All annotated members of the Krüppel-like factor family (KLF1–KLF17) were included without pre-selection bias.

Comparative analyses focused on “Heart—Left Ventricle” tissue samples as representative cardiac tissue and “Cells—Cultured fibroblasts” (human dermal fibroblasts) as a non-cardiac reference population. For each tissue, TPM values were averaged across all donors to generate representative tissue-level expression profiles. Heatmaps were generated using relative expression scaling to facilitate visualization of tissue-specific expression patsterns.

### 2.6. Protein–Protein Interaction Analysis

Protein–protein interaction networks were constructed using STRING (version 11.5). Genes analyzed in this study were included to identify potential regulatory interactions. The analysis included selected KLF family members together with genes associated with cardiac remodeling, inflammation, fibrosis, electrical conduction, calcium handling, and signaling pathways identified through RT-qPCR analysis and complementary transcriptomic exploration.

Networks were generated based on experimental evidence, co-expression, curated databases, and text mining. Interactions were analyzed using the default STRING interaction settings, and network enrichment statistics were obtained directly from the platform. KLF family members were organized according to previously described structural subgroup classifications based on conserved zinc-finger domain homology and transcriptional regulatory characteristics.

Functional enrichment analyses were performed within STRING to identify overrepresented biological processes, molecular functions, signaling pathways, and disease-associated terms linked to the interaction network. Attention was given to pathways associated with inflammatory signaling, TGF-β/SMAD signaling, electrophysiological regulation, and cardiac remodeling.

The resulting interactome was interpreted as an exploratory framework to identify potential regulatory associations and signaling convergence points; STRING-derived interactions should not be interpreted as evidence of direct mechanistic regulation.

### 2.7. Microarray Data Analysis

Publicly available microarray data were obtained from the Gene Expression Omnibus (GEO) repository through the dataset GSE18801, “Transcriptional profile of isoproterenol-induced cardiomyopathy and comparison to exercise-induced cardiac hypertrophy.” The dataset corresponds to murine cardiac tissue subjected to isoproterenol-induced hypertrophy and includes control and experimental groups analyzed using the Affymetrix Mouse Genome 430 2.0 Array platform.

Differential expression analysis was performed using the GEO2R online analysis tool provided by the National Center for Biotechnology Information (NCBI). Comparisons were conducted between isoproterenol-treated and control samples within the dataset. Statistical analysis within GEO2R was based on the limma package version 3.60.6, and adjusted *p* values were calculated using the Benjamini–Hochberg false discovery rate correction method as implemented by the GEO2R platform.

Volcano plot visualization was generated using relative fold-change and statistical significance values obtained from GEO2R to identify genes exhibiting differential expression patterns within the dataset. Given the exploratory and comparative nature of this analysis, no additional independent filtering pipeline or external normalization procedures were applied beyond those incorporated within the GEO2R workflow.

Genes associated with KLF signaling, inflammatory mediators, cardiac transcription factors, calcium handling, and electrophysiological regulation were qualitatively examined for comparison with observations obtained in the present rat remodeling model.

The GEO analysis was used as a complementary transcriptomic reference to contextualize temporal expression patterns identified in the experimental model. 

### 2.8. Statistical Analysis

Data are presented as mean ± SEM. Statistical analyses were performed using SPSS version 17.0 (SPSS Inc., Chicago, IL, USA). Graphical representations were generated using GraphPad Prism (version 10.2.0). Schematic illustrations were prepared using BioRender.com Version 10.2.0.

Comparisons between experimental groups and the shared control group across all time points were analyzed using one-way ANOVA followed by Dunnett’s post hoc test for multiple comparisons. A *p* value of < 0.05 was considered statistically significant.

For RT-qPCR analyses, data were derived from three biological replicates per time point, with two technical replicates analyzed per sample. Immunohistochemical observations were interpreted descriptively and qualitatively.

Given the exploratory and temporal nature of the study, transcriptomic integration analyses derived from GTEx, GEO, and STRING were interpreted as comparative approaches rather than definitive mechanistic validation.

## 3. Results

### 3.1. Morphological and Molecular Characterization of Isoproterenol-Induced Myocardial Remodeling

A single intraperitoneal administration of isoproterenol (65 mg/kg) was used to induce myocardial injury in Wistar rats, which were monitored over a 21-day period encompassing the classical phases of cardiac remodeling: early inflammatory (days 1–4), proliferative (days 5–14), and maturation (days 15–21).

**Figure 1:**
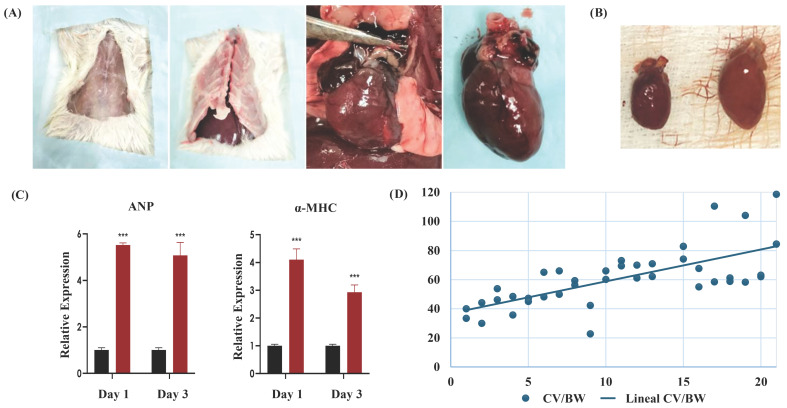
Isoproterenol-induced cardiac injury and hypertrophic remodeling. (**A**) Representative images illustrating the surgical procedure for heart exposure and excision. (**B**) Gross morphology of excised hearts, showing a representative control heart (left) and a heart collected 21 days after isoproterenol administration (right), evidencing cardiac enlargement. (**C**) Relative gene expression of atrial natriuretic peptide (ANP, cardiac stress marker) and α-myosin heavy chain (α-MHC, hypertrophic marker) assessed by qRT-PCR in control (black) and isoproterenol-treated rats at Day 1 and Day 3 post-administration (red). Gene expression levels were normalized to GAPDH and expressed as mean ± SEM. *** *p* < 0.001 vs. control. (**D**) Cardiac volume, calculated using the formula V = (**3**/**4**) ***π*** × ***D*1** × ***D*2** × ***L*** and normalized to body weight (*y*-axis), plotted across the experimental time course (*x*-axis) in control and treated rats. Statistical comparisons were performed using one-way ANOVA followed by Dunnett’s post hoc test (two-sided). *** *p* < 0.001 vs. control.

Representative images of the cardiac excision procedure and tissue processing are shown in Figure 1A. At a macroscopic level, isoproterenol-induced injury-treated hearts showed progressive enlargement compared to the controls, with clear hypertrophy visible at the late time points (Figure 1B). Morphometric analysis revealed that cardiac volume normalized to body weight (CV/BW) increased steadily throughout the remodeling period. By day 7, the CV/BW ratio was approximately 1.3-fold higher compared to the baseline controls, and by day 21, this increase reached nearly 2-fold (Figure 1D). These results indicate progressive structural remodeling over the experimental period.

At the molecular level, cardiac stress markers showed early and sustained changes. Atrial natriuretic peptide (ANP) expression increased approximately 5-fold at 24 h, while α-myosin heavy chain (α-MHC) increased approximately 4-fold at 24 h and remained elevated at ~3-fold at 72 h relative to controls (Figure 1C). These changes are consistent with the activation of a cardiac stress response following isoproterenol administration.

Histological analyses corroborated pathological myocardial remodeling. Hematoxylin and eosin (H&E) staining revealed myocardial fiber disorganization and increased interstitial spacing in isoproterenol-treated hearts compared with controls. (Figure S1A–D).

As observed, our initial results confirm that isoproterenol-induced cardiac injury led to progressive structural remodeling and early activation of cardiac stress markers, histological disorganization, and temporally regulated changes in remodeling-associated transcriptional and calcium-handling proteins.

### 3.2. Baseline Gene Expression Patterns in Human Cardiac and Non-Cardiac Tissue

To contextualize the experimental findings, baseline gene expression profiles were analyzed using human data from the Genotype-Tissue Expression (GTEx) project. Expression profiles from the human left ventricle (LV) were compared with cultured skin fibroblasts as a non-cardiac reference cell type.

Gene expression profiles were analyzed using publicly available GTEx datasets, with expression values reported as transcripts per million (TPM). KLF family members and cardiac- and inflammation-related genes were compared between left ventricular tissue and non-cardiac fibroblasts using heatmap-based visualization to highlight tissue-associated expression patterns.

**Figure 2:**
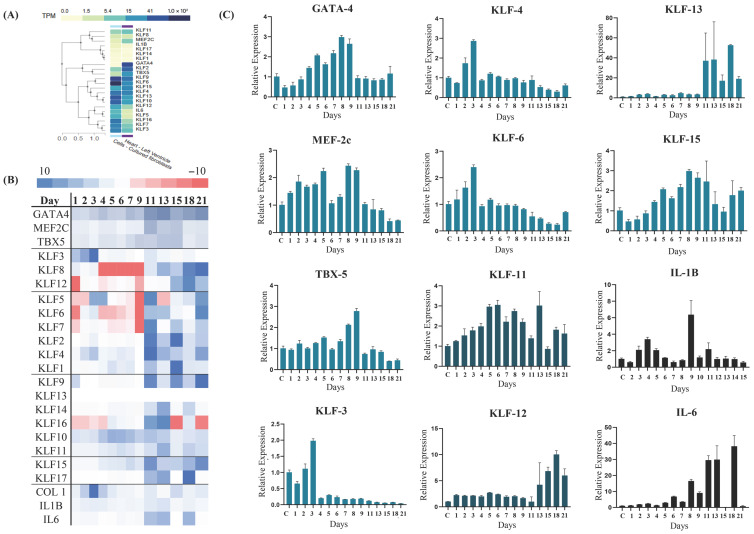
Temporal transcriptional dynamics of Krüppel-like factors, cardiac remodeling markers, and inflammatory genes following isoproterenol-induced cardiac injury. (**A**) Heatmap of KLF family gene expression and related genes in normal human heart tissue (TPM), compared to fibroblasts as a noncardiac control. (**B**) Temporal heatmap showing relative expression patterns of selected KLFs and inflammatory genes across different days after heart injury, illustrating early-, mid-, and late-stage remodeling phases. (**C**) RT–qPCR analysis of representative remodeling-associated genes, KLFs, and inflammatory markers. The *x*-axis denotes days after isoproterenol administration, and the *y*-axis represents relative gene expression normalized to GAPDH.

Analysis revealed a distinct tissue-specific expression pattern. Several KLFs, including KLF4 and KLF15, were highly enriched in the human LV, consistent with cardiomyocyte homeostasis and stress adaptation [16,17]. In contrast, KLF9 and KLF13 showed relatively higher expression in fibroblasts, aligning with their association with stress and remodeling-related transcriptional programs described in non-cardiac contexts [18,19,20]. Finally, KLF5 exhibited a fibroblast-enriched pattern, in line with its reported association with proliferative and pathological remodeling states, including heart failure and diabetic cardiomyopathy [12,15] (Figure 2A).

Basal expression of inflammatory mediators IL-6 and TNF-α was low in healthy cardiac tissue but comparatively higher in fibroblasts, in line with the recognized role as primary contributors to paracrine inflammatory signaling. Importantly, the cardiac transcriptional triad GATA4, MEF2C, and TBX5 was markedly enriched in the LV relative to the fibroblasts.

### 3.3. Temporal Expression Dynamics of KLFs, GMT, and Inflammatory Mediators

Following isoproterenol treatment, rat heart gene expression analysis revealed dynamic and phase-specific changes (Figure 2B,C). During the early inflammatory phase (days 1–4), KLF4 showed a marked increase (3-fold relative to the control at days 2–3), coinciding with elevations of KLF3 and KLF6 (peaking ~2-fold and ~2.5-fold at day 3). These changes coincided with early rises in IL-1β and IL-6, in accordance with activation of an inflammatory response.

**Figure 3:**
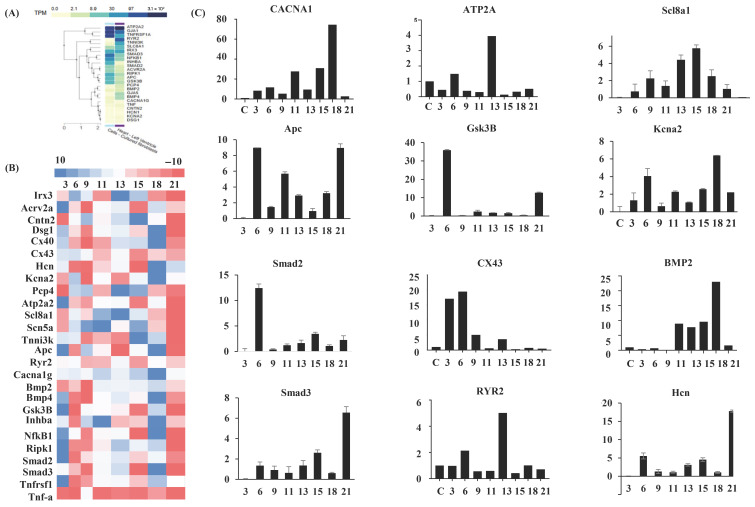
Temporal transcriptional profiling of cardiac electrical conduction and excitation–contraction coupling genes during isoproterenol-induced cardiac remodeling. (**A**) Heatmap of electrical and excitation–contraction coupling genes in normal human heart tissue (TPM). (**B**) Relative expression changes in genes involved in electrical signaling, gap junction communication, and selected regulatory and inflammatory markers across different days following cardiac injury. (**C**) RT–qPCR validation of representative cardiac regulatory, electrical, and inflammatory genes. Relative gene expression was normalized to GAPDH.

During the proliferative stage (days 5–14), KLF11 expression, a known regulator of TGF-β/SMAD signaling pathway [18,19,20], peaked at days 5–6 (~3-fold increase), and SMAD3 showed a temporal increase on days 6–21 (Figure 3B,C). By day 8, KLF15 showed a strong upregulation (~3-fold), temporally coinciding with a progressive decline in the Gata4, Mef2c, and Tbx5 (GMT) expression toward near-basal levels after day 9 [21]. An inverse temporal relationship was observed between Klf15 expression and GMT-associated gene expression.

During the maturation phase (days 15–21), KLF12 and KLF13, factors are associated with pro-apoptotic signaling and fibrosis [22,23,24]. Notably, IL-6 levels remained elevated throughout all stages, suggesting persistent low-grade inflammation (Figure 2C).

To complement these findings, publicly available microarray data from the Gene Expression Omnibus (GEO) were analyzed using dataset GSE18801 to explore transcriptional changes associated with isoproterenol-induced cardiac injury. Differential expression analysis was visualized using a volcano plot (Figure S2), which demonstrated a mixed pattern of up- and downregulated genes consistent with a stress-responsive transcriptional program rather than a unidirectional expression shift.

Among the differentially expressed genes, increased expression of Klf3, Klf9, Klf12, and Klf15 was observed along with changes in cardiac and signaling-related genes, including Cacna1c, Gata4, Mef2c, Ryr2, Atp2a2, Tnni3k, Pcp4, and Tnfrsf1a. Several of these genes overlapped with those identified in the network-based analysis, suggesting partial overlap in pathway-level signals across datasets in pathways related to transcriptional regulation, inflammatory signaling, and excitation–contraction coupling.

### 3.4. STRING Interactome Analysis

**Figure 4:**
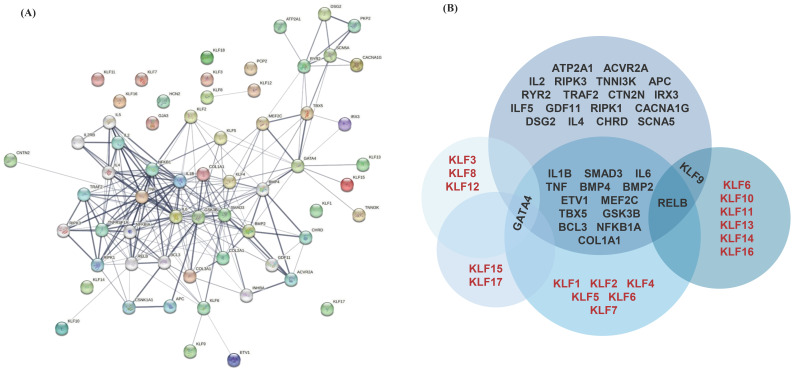
Protein–protein interaction network of Krüppel-like factor (KLF) family members and associated cardiac regulatory genes. (**A**) Protein–protein interaction network generated using the STRING database (version 11.5), showing predicted and experimentally derived associations among KLFs and genes associated with cardiac hypertrophy, fibrosis, inflammatory signaling, and electrical conduction. Nodes represent proteins, and edges indicate known or predicted interactions based on experimental evidence, co-expression, gene neighborhood, text mining, and curated database annotations. (**B**) Venn diagram illustrating the overlap of genes associated with distinct KLF family members, grouped according to their structural classification: subgroup 1 (KLF3, KLF8, KLF12), subgroup 2 (KLF1, KLF2, KLF4, KLF5, KLF6, KLF7), subgroup 3 (KLF9, KLF10, KLF11, KLF13, KLF14, KLF16), and ungrouped members (KLF15 and KLF17).

Protein–protein interaction networks were constructed using STRING (version 11.5) to explore potential functional associations among KLF family members and genes implicated in cardiac remodeling, inflammation, and electrophysiological regulation (Figure 4). The analysis integrated known and predicted interactions derived from experimental evidence, curated databases, co-expression, gene neighborhood, and text mining.

Our screening revealed associations of components related to cardiac electrical function (Na/K pumps, calcium exchangers), as well as Wnt, BMP, SMAD signaling elements, and inflammatory mediators; key components included Irx3, Acrv2a, Cntn2, Dsg1, Cx40, Cx43, Hcn, Kcna2, Pcp4, Atp2a2, Scl8a1, Scn5a, Tnni3k, Apc, Ryr2, Cacna1g, Bmp2, Bmp4, Gsk3B, Inhba, NfkB1, Ripk1, Smad2, Smad3, Tnfrsf1, and Tnf-a. KLF family members were distributed across functional clusters, indicating connectivity with genes involved in stress response and cardiac remodeling pathways.

To further organize these interactions, KLF family members were grouped according to their structural classification, showing distinct interaction patterns across subfamilies. KLF-associated gene clusters overlapped with inflammatory, transcriptional, and electrophysiological pathways, suggesting shared functional environments within cardiac remodeling.

The full list of STRING-derived interactions, including confidence scores and functional annotations, is provided in Table S3. This dataset complements the network visualization (Figure 4) by enabling a detailed inspection of individual KLF-associated interaction partners and supporting pathway-level interpretation. Importantly, STRING-derived associations represent integrated evidence from experimental data and computational predictions.

## 4. Discussion

### 4.1. Phase-Associated Expression of KLFs During Cardiac Remodeling

The heart, a vital organ responsible for systemic oxygen and nutrient delivery, exhibits limited regenerative capacity, rendering it highly susceptible to pathological remodeling following myocardial injury [6,25,26,27]. Although cardiac hypertrophy initially serves as a compensatory response to preserve function, its progression is governed by complex transcriptional and signaling networks that ultimately contribute to maladaptive remodeling and heart failure [10]. Despite the high prevalence of myocardial infarction, the regulatory mechanisms coordinating these transitions remain incompletely understood.

In this context, we employed an isoproterenol-induced cardiomyopathy model to examine transcriptional regulation during cardiac remodeling. The KLF family represents a functionally diverse group of transcription factors implicated in development, stress responses, and cardiovascular disease [6,28,29,30]. This model reproduces key features of myocardial injury, including fibrosis, cardiomyocyte loss, and hypertrophic remodeling [31,32], providing a framework to investigate phase-associated KLF expression programs.

Our data show that KLF expression exhibits phase-specific patterns during cardiac remodeling. In the early inflammatory phase (days 1–4), we observed a rapid upregulation of Klf4, Klf3, and Klf6 with peak expression around day 3. These early transcriptional changes coincided with increased expression of inflammatory mediators, such as Il-1β and Il-6, consistent with activation of the inflammatory response following isoproterenol-induced injury. Previous studies have associated several KLFs with stress-responsive, pro-fibrotic, and anti-apoptotic signaling pathways during cardiovascular remodeling [26].

Our interactome analysis identified associations between Klf6, Il-6, and components of the NF-κB pathway, including Nf-κb1a and Relb [33]. In addition, Klf6 showed connectivity with Gata4 and Smad3, suggesting potential association with transcriptional programs associated with TGF-β/SMAD signaling, a contributor of fibrotic remodeling and collagen gene induction during scar formation [18].

The subsequent proliferative phase revealed a potential transition point in the remodeling transition between remodeling phases. During this phase, we observed robust increases in both Klf11 and Klf15 with a 3-fold increase relative to control. Klf11 has been previously described as a downstream effector of TGF-β signaling capable of cooperating with SMAD transcription factors to modulate TGF-β–dependent transcriptional programs [19,20,34]. In line with this, we detected increased expression of Smad3, along with elevated Bmp2, a member of the TGF-β superfamily, suggesting engagement of TGF-β–related signaling networks commonly associated with fibrotic remodeling [18].

Notably, the peak expression of Klf15 around day 8 was associated with the downregulation of the cardiac commitment triad GMT. This inverse relationship is consistent with previously described roles of Klf15 as a negative regulator of pathological hypertrophy [35,36,37]. This mechanism is supported by previous studies showing that Klf15 directly blocks Gata4 and Mef2c DNA-binding sites, thereby limiting pro-hypertrophic gene expression of Myocardin [35,38,39]. Although our study was not designed to establish direct regulatory causality, the temporal association observed between Klf15 induction and declining GMT expression suggests coordinated transcriptional changes across remodeling phases.

This antagonistic role is associated with a later remodeling stage characterized by an upregulation of pro-apoptotic regulators Klf12 and Klf13 (2–3-fold), which likely contribute to scar consolidation and the final patterning of the myocardium [23,40]. In fact, murine models of coronary artery ligation have shown an increase in Klf13 related to early cardiomyocyte death, a contrasting effect to the anti-apoptotic role of Klf4 [30,40].

### 4.2. GMT Factors, Cardiac Conduction-Associated Genes, and Calcium Handling

The GMT genes are well-established for their critical roles in cardiac development, contractility, stress adaptation, and hypertrophic remodeling [39,41,42,43,44]. In the present study, expression analysis revealed phase-associated regulation of GMT-associated genes during isoproterenol-induced remodeling. Although several GMT-related factors exhibited early increases following injury, reduced expression during later remodeling stages coincided with broader transcriptional and structural changes within the myocardium.

These changes were accompanied by altered expression of genes associated with excitation–contraction coupling and cardiac electrical regulation, including Ryr2, Atp2a2, Cx43, and Slc8a1. Reduced expression of calcium-handling genes such as Atp2a2 and Ryr2 during intermediate and late remodeling stages may reflect impaired calcium cycling and contractile dysfunction commonly associated with pathological remodeling states. Similarly, changes in Cx43 and Slc8a1 expression suggest remodeling-associated adaptations involving gap junction communication and ionic homeostasis during myocardial stress.

Previous research has shown that cardiac injury and hypertrophic agonists activate MAPK signaling cascades, including p38 and ERK1/2, both of which participate in the regulation of GATA4-dependent hypertrophic transcription [45,46,47]. Activation of these pathways has been associated with increased GATA4 transcriptional activity and induction of hypertrophic gene programs during cardiac stress. Experimental models have further demonstrated that Gata4 overexpression can promote hypertrophic remodeling in vivo [47]. Our findings also suggest that remodeling-associated transcriptional changes extend beyond hypertrophic signaling and involve genes associated with cardiac conduction and calcium handling. Previous studies have linked TBX5 with SRF- and CX43-associated transcriptional regulation, both of which participate in myocardial stress adaptation and electrical coupling within cardiac tissue [43,44]. In parallel, our interactome analysis identified network associations between multiple KLF family members and genes related to conduction, calcium handling, and stress-responsive transcriptional programs, including Gata4, Cx43, Atp2a2, Slc8a1, and Ryr2. Additional associations involving Klf6, Gata4, and Smad3 further suggest potential overlaps between inflammatory, hypertrophic, and fibrosis-associated signaling environments during remodeling progression.

### 4.3. Remodeling Patterns and Integration with Transcriptional Findings

KLF4 demonstrated a modest reduction during early remodeling stages, followed by increased around day 14, highlighting the complexity of post-transcriptional regulation. Previous studies have associated KLF4 with stress and fibrosis, the upregulation of TGF-β1 signaling [48,49]. In this context, the delayed increase in KLF4 expression relative to early transcriptional activation may reflect temporally regulated remodeling responses occurring during the proliferative phase. In parallel, our transcriptional findings showing increased expression of Col1A1, Col3A1, and SMAD-associated genes are consistent with the activation of fibrosis-associated remodeling pathways during this stage.

The increase in KLF15 by day 8 observed in PCR is consistent with the early remodeling stage. KLF15 is a negative regulator of hypertrophy, mostly through Gata4 and Mef2c interactions [49]. Hence, our results show a temporal relationship between KLF15 induction and declining GMT expression, supporting the possibility of coordinated transcriptional remodeling during disease progression.

Interestingly, although TBX5 remained relatively stable throughout the remodeling period, RT-qPCR analyses demonstrated dynamic changes in several conduction and calcium handling genes, including Cx43 and Slc8a1. Rather than directly indicating preserved conduction system integrity, these findings may suggest that downstream or associated transcriptional pathways continue to undergo remodeling-associated adaptation despite relatively stable TBX5 protein localization within myocardial tissue.

## 5. Study Limitations

The experimental model was conducted exclusively in female Wistar rats, which may limit generalizability given known sex-dependent differences in cardiac remodeling, inflammation, and fibrosis. Future studies including both sexes will be required to determine whether the observed KLF-associated temporal patterns are sex-dependent.

The study design is descriptive and time-resolved rather than mechanistically interventional. While strong temporal associations were identified among KLF family members, GMT factors, and calcium-handling genes, causal regulatory relationships cannot be inferred. Functional validation will be required to confirm directionality within the proposed networks.

Bioinformatic analyses (STRING, GTEx, and GEO datasets) were used for contextual and integrative interpretation. These approaches are dependent on curated and predictive frameworks and should therefore be considered hypothesis-generating rather than evidence of direct biological interaction.

Finally, the study focuses on a 21-day window, capturing early to intermediate remodeling phases but not long-term progression toward advanced heart failure phenotypes.

## 6. Conclusions

Our integrated transcriptomic, bioinformatic, and protein-level analyses provide a temporal framework of isoproterenol-induced cardiac remodeling, highlighting the coordinated regulation of KLFs across distinct phases of injury and repair.

Distinct KLF family members showed phase-associated expression patterns. During the early inflammatory phase, Klf4, Klf3, and Klf6 were prominently upregulated, coinciding with increased expression of proinflammatory cytokines, supporting their association with early stress-responsive and profibrotic remodeling. In the proliferative phase, Klf11 and Klf15 were most prominently expressed, aligning with the activation of TGF-β/SMAD-related pathways and modulation of cardiac transcriptional programs. The temporal association between Klf15 upregulation and the decline in GATA4, MEF2C, and TBX5 suggests a potential role in attenuating pro-hypertrophic signaling during this transition. In the maturation phase, Klf12 and Klf13 were upregulated, consistent with processes associated with fibrosis, apoptosis, and scar stabilization. These patterns collectively support a model in which KLFs act in a temporally coordinated manner to regulate inflammatory, structural, and transcriptional responses during cardiac remodeling.

These findings provide an integrated framework linking experimental and publicly available datasets and support further investigation into the mechanistic role of individual KLFs in cardiac remodeling.

## Data Availability

The original contributions presented in this study are included in the article/Supplementary Materials. Further inquiries can be directed to the corresponding author.

## Supplementary Materials

The following supporting information can be downloaded at https://www.mdpi.com/article/10.3390/genes17060657/s1. Supplemental Figure S1. Histological and immunohistochemical characterization of isoproterenol-induced cardiac remodeling. (A–H) Representative hematoxylin and eosin (H&E) staining of myocardial tissue showing structural alterations, interstitial expansion, and myocardial fiber disorganization in isoproterenol-treated hearts compared with controls. (A,C) At 10X, 21d post infarct high fibroblast infiltration, (B,D) Corresponding 40X, 21d post infarct, (E) 10X Loss of muscle fibers, (F) Corresponding 40X Loss of muscle fibers, (G) 10X Control no infarct, (H) 40X Control o infarct. Supplemental Figure S2. Volcano plot for 4498 differentially expressed genes in GSE 18801. Differential expression analysis comparing isoproterenol-induced cardiomyopathy vs. control of the left ventricle using Affymetix Mouse Genome 430 2.0 Array. Supplemental Table S1. Weights (g) and lengths (mm). Supplemental Table S2. Primer list. Supplemental Table S3. Table of protein–protein interaction pairs from the STRING database.

## Abbreviations

CTRL	Control
CV/BW	Cardiac Volume Normalized to Body Weight
DEG	Differentially Expressed Genes
GEO	Gene Expression Omnibus
GTEx	Gene-Tissue Expression
H&E	Hematoxylin and Eosin
IHC	Immunohistochemistry
KLF	Krüppel-Like Factors
