# Supplementary material for: Temporal Orchestration of Krüppel-like Factors During Cardiac Remodeling Following Isoproterenol-Induced Myocardial Injury
Source: Genes (Basel). 2026 Jun 3;17(6):657. doi: 10.3390/genes17060657 (PMC13299128; doi:10.3390/genes17060657)
Supplement: Supplementary file 1 [file genes-17-00657-s001.zip › Supplementary Table S1.pdf]

Supplementary Table S1. Table of weights and lengths*

0	Body Weight** (g)	Cardiac Weight** (g)	Length ** (mm)	Diameter ** (mm)
1	191	1	14.8	11.1
	245	1	13.6	12
2	240	1	17.6	12
	184	1	14	9.7
3	177	1	18.4	10.3
	170	1	18.4	10.9
4	183	0.8	14.7	12
	170	1	14.5	10
5	187	0.9	17.1	11.1
	181	0.8	17	10.7
6	207	0.8	15	12.6
	221	1	18.3	13.7
7	201	0.8	15.6	12.4
	203	1	17.3	13.6
8	193	1	16.1	12.7
	199	0.9	12.2	15.2
9	216	0.8	11.5	10.1
	188	0.7	13.2	12
10	160	0.8	16.4	12.4
	282	2	18.5	14.8
11	207	1.1	21.3	12.7
	218	1	20.3	13.7
12	196	0.9	17.2	13.8
	210	1	17.6	13.2
13	193	0.8	11.2	16
	178	1	16.8	13.4
14	221	0.9	15.6	11.4
	192	0.6	13.4	12
15	166	1	16.3	14.2
	205	1.1	12	17.4
16	184	1.1	16	12.3
	210	1	18.1	13.7
17	186	0.7	16.9	12.4
	199	1.2	20.5	16
18	279	2	20	14

	187	2	19	12
19	207	2	20	12
	208	2	20.2	16
20	206	1	17	13.4
	251	2	18	14.5
21	184	1	10.5	18.8
	175	2	15.3	18

* Repeat runs

**Statistical values rounded (n = 3)
