# Supplementary material for: Temporal Orchestration of Krüppel-like Factors During Cardiac Remodeling Following Isoproterenol-Induced Myocardial Injury
Source: Genes (Basel). 2026 Jun 3;17(6):657. doi: 10.3390/genes17060657 (PMC13299128; doi:10.3390/genes17060657)
Supplement: Supplementary file 1 [file genes-17-00657-s001.zip › Supplementary Table S2.pdf]

Supplementary Table S2. List of primers.

GENE	5´FWD	5´REV	Reference (Accesion /Ensembl)	Location (Exons)
Acrv2a	GAGGTGTTAGAGGGTGCTATA	GAGGTGTTAGAGGGTGCTATA	ENSRNOT00000007404.8	10-12
Apc	TTCAGGAAAACGACAATGGGA	CTTTTTGGCTTTGCGTGATGA	ENSRNOT00000090264.2	16
Atp2a2	CAAAGGCTTTTACAGGGCGAG	CACCATCACCAGTCATAGCTG	ENSRNOT00000024347.8	15-16
Bmp2	GGGACCCGCTGTCTTCTAGT	TCAACTCAAACTCGCTGAGGAC	NM_007553	2
Bmp4	GGAAGAAGAGCAGAGCCAGGG	TTCTCTGGGATGCTGCTGAGG	ENSRNOT00000012957.7	3-10
Cntn2	GACACCTACTCCCAACAGTAC	TCCAGGCATTAGGCCTAGTAC	ENSRNOT00000012190.4	23
Col1A1	GCTCCTCTTAGGGGCCACT	GGGTCCCTCGACTCCTATGA	NM_007742	1-2
Cx43	ACAGCTGTTGAGTCAGCTTG	GAGAGATGGGGAAGGACTTGT	NM_010288	2
Dsg	CAGTTTTATCGAGGGAGAGCT	TGTTTCTCTGACCGGCTTTTG	ENSRNOT00000103330.1	14
Gapdh	AGGTCGGTGTGAACGGATTTG	TGTAGACCATGTAGTTGAGGTCA	NM_008084	2-3
Gata 4	CCCTACCCAGCCTACATGG	ACATATCGAGATTGGGGTGTCT	NM_008092	1-2
Gja5	GGGCTACCACAGTGACAAGCG	CCGGGAGAGAAGGTGCTGAGG	ENSRNOT00000115241.1	2
Gsk3B	GTGGCGAGAAGAAAGATGAGG	GATGGCAGATCCCAAAGGAAT	ENSRNOT00000003867.7	3-6
Hcn	TTTCGCACCGGCATTGTTATT	CTCCACGATGAGGAAGATGTA	ENSRNOT00000011837.7	2-3
Inhba	AGAAAGTGGTAGATGCTCGGA	CTTTTTCCTCTTCCAGCCCTC	ENSRNOT00000019272.6	3
Irx3	AGAGGAGTCAGACTAGGAAAA	GCAGTTCTGGACGCTGGGAAA	ENSRNOT00000015583.6	1
Kcna2	GACACAGTGGCTTAAATTCCC	ATATGGGTGACCAGGAACTAA	ENSRNOT00000116262.1	3
Klf-1	AGACTGTCTTACCCTCCATCAG	GGTCCTCTGATTTCAGACTCAC	NM_010635	1-2
Klf-10	ATGCTTAACTTCGGCGCTT	CGCTTCCACAGCTTCAAAG	NM_013692	1-2
Klf-11	CATGGACATTTGTGAGTCAATCC	CCTTTGGTAGATCAGGTGCAG	NM_178357	2
Klf-12	CAAGGGTCTCCAAACGTCCAC	TGATCTACAGGCAATGAGTCC	NM_010636	4
Klf-14	CTCCGTGTGCCTCAACAAGCTGC	CAGGCGCACCCAGGATAGC	NM_001135093	1
Klf-15	GAGACCTTCTCGTCACCGAAA	GCTGGAGACATCGCTGTCAT	NM_023184	2
Klf-16	ATCCTGGCCGATCTGAGAGG	GTGCGAAGACTTGTAATAGGCT	NM_078477	1
Klf-17	TTCCTGTGGAGTCTCAGTGAT	CGAGTGCAACATCTGATTCATA	NM_029416	2

Klf-2	CTCAGCGAGCCTATCTTGCC	CACGCTGTTTAGGTCCTCATCC	NM_008452	1-2
Klf-3	GAAGCCCAACAAATATGGGGT	GACAAGGAGACCATGAGAGG	NM_008453	3
Klf-4	GTGCCCCGACTAACCGTTG	TCGTTGAACTCCTCGGTCT	NM_010637	2-3
Klf-5	CCGGAGACGATCTGAAACACG	GTTGATGCTGTAAGGTAAGCCT	NM_009769	1-2
Klf-6	GTTTCTGCTCCGACTCCTGAT	TTCCTGGAAGATGCTACACATTG	NM_011803	1
Klf-7	TCCACGACACCGGCTACTT	GGGAGCAGCAAGGGGTCTA	NM_033563	1-2
Klf-8	GATTCGATGGAGGTATTCAAA	AACAGAGCTGGGTTCTCCATT	NM_173780	3-4
Klf-9	GCCGCCTACATGGACTTCG	GCCGTTCACCTGTATGCAC	NM_010638	1-2
Mef 2c	ATGCCATCAGTGAATCAAAGGAT	GTGGTACGGTCTCCCAACT	NM_001170537.2	8-11
NfkB1	GAAAATGGTGGAGTTTGGGAA	CTCCTTGTCTTTGATTTCGGG	ENSRNOT00000109057.1	10-12
Nppa	GCTCCCAGGCCATATTGGAG	GGAGGCATGACCTCACTTC	NM_012612.2	2
Pcp4	GTGCTGGGGCAACCAATGGAA	CTCTGGTGCATCCATGTCGAT	ENSRNOT00000002221.7	2
Ripk1	CTAGCTTCCCACTTCATGTAC	TACCTGAAGATCTCTCTCTGC	ENSRNOT00000023962.6	11
Ryr2	ACCGCAACCATCCACAAAG	AAAGTCTGTTGCCAAATCCTCCT	NM_023868	2
Scn5a	ACAACTTCAACCAGCAGAAGA	GATGGGCTTCTGGGGTTTCTT	ENSRNOT00000082892.2	25-27
Slc8a1	CCGTCTGGTGGAGATGAGTGA	CTCTCCTCCTCCTCTTTGCTG	ENSRNOT00000042230.6	3-10
Smad2	CAATCACAGCTTGGATTTGCA	AACGTTGGAGAGCAAACCTAA	ENSRNOT00000092173.2	7-10
Smad3	CAGGACTGGAGTGTGGAGTTC	TCCAAGGAAGAAGCGGTTCTC	ENSRNOT00000039730.5	9
Tbx5	AATGGTCCGTAACTGGTAAAG	GGATAATGTGTCCGAATGGGTC	NM_011537	4-6
Tnf-B	CCACCTCCTGAGGGTGCTTAGC	GCCATGTCGGAGAAAGGCGC	NM_010735	2-4
Tnfrsf1	TTCTCCTTGCAAAGCTGACAT	GTGCACTCATTTCCGCTTAGA	ENSRNOT00000102042.1	3-6
Tnni3k	GGAAATTTTGAAGTTGCCAAG	AGAAATTTGACCAGGTCAATG	ENSRNOT00000120189.1	9-16
Tnfrsf1	TTCTCCTTGCAAAGCTGACAT	GTGCACTCATTTCCGCTTAGA	ENSRNOT00000102042.1	3-6
