# Supplementary material for: Temporal Orchestration of Krüppel-like Factors During Cardiac Remodeling Following Isoproterenol-Induced Myocardial Injury
Source: Genes (Basel). 2026 Jun 3;17(6):657. doi: 10.3390/genes17060657 (PMC13299128; doi:10.3390/genes17060657)
Supplement: Supplementary file 1 [file genes-17-00657-s001.zip › Supplementary Table S3.pdf]

#node1	node2	neighborhood_on_chromosome	gene_fusion	phylogenetic_cooccurrence	homology	coexpression	experimentally_determined_interaction	database_annotated	automated_textmining	combined_score
ACVR2A	CHRD	0	0	0	0	0	0.047	0	0.49	0.493
ACVR2A	SMAD3	0	0	0	0	0.086	0.126	0.9	0.768	0.979
ACVR2A	BMP4	0	0	0	0	0.048	0.364	0.9	0.963	0.997
ACVR2A	GDF11	0	0	0	0	0.048	0.126	0.9	0.994	0.999
ACVR2A	INHBA	0	0	0	0	0.048	0.982	0.9	0.979	0.999
ACVR2A	BMP2	0	0	0	0	0.05	0.973	0.9	0.876	0.999
APC	CSNK1A1	0	0	0	0	0.05	0.292	0.8	0.994	0.999
APC	GSK3B	0	0	0	0	0.095	0.863	0.9	0.996	0.999
ATP2A1	RYR2	0	0	0	0	0.11	0.103	0	0.527	0.59
BCL3	TNFRSF1A	0	0	0	0	0.276	0	0	0.351	0.51
BCL3	COL3A1	0	0	0	0	0.056	0.095	0	0.383	0.427
BCL3	KLF6	0	0	0	0	0.081	0.091	0	0.441	0.492
BCL3	TNF	0	0	0	0	0.163	0	0	0.473	0.54
BCL3	KLF4	0	0	0	0	0.154	0.091	0	0.465	0.552
BCL3	GSK3B	0	0	0	0	0.042	0.331	0	0.528	0.671
BCL3	IL1B	0	0	0	0	0.213	0	0	0.657	0.719
BCL3	IL6	0	0	0	0	0.176	0	0	0.721	0.761
BCL3	NFKBIA	0	0	0.186	0.681	0.257	0	0.72	0.5	0.904
BCL3	RELB	0	0.105	0	0	0.475	0.344	0	0.882	0.959
BCL3	NFKB1	0	0	0	0.662	0.175	0.835	0.9	0.984	0.999
BMP2	CHRD	0	0	0	0	0	0.474	0.9	0.985	0.999
BMP2	COL1A1	0	0	0	0	0.068	0.099	0	0.662	0.691
BMP2	ACVR2A	0	0	0	0	0.05	0.973	0.9	0.876	0.999
BMP2	BMP4	0	0	0.055	0.96	0.072	0	0.9	0.708	0.97
BMP2	RIPK1	0	0	0	0	0.044	0.126	0	0.392	0.447
BMP2	IL1B	0	0	0	0	0.08	0	0	0.588	0.605
BMP2	GSK3B	0	0	0	0	0	0.126	0	0.465	0.512
BMP2	SMAD3	0	0	0	0	0	0.161	0	0.587	0.638

BMP2	KLF4	0	0	0	0	0.065	0.054	0	0.386	0.409
BMP2	GATA4	0	0	0	0	0.042	0.07	0	0.474	0.49
BMP2	IL6	0	0	0	0	0.091	0	0	0.576	0.599
BMP2	TNF	0	0	0	0	0.058	0	0	0.726	0.731
BMP2	COL2A1	0	0	0	0	0	0.334	0	0.792	0.855
BMP4	CHRD	0	0	0	0	0	0.474	0.9	0.991	0.999
BMP4	COL1A1	0	0	0	0	0.083	0.099	0	0.532	0.579
BMP4	ACVR2A	0	0	0	0	0.048	0.364	0.9	0.963	0.997
BMP4	TNF	0	0	0	0	0.056	0	0	0.444	0.452
BMP4	KLF2	0	0	0	0	0	0.054	0	0.445	0.453
BMP4	IL1B	0	0	0	0	0.047	0	0	0.457	0.461
BMP4	MEF2C	0	0	0	0	0	0	0	0.529	0.529
BMP4	COL2A1	0	0	0	0	0.042	0.099	0	0.5	0.531
BMP4	GSK3B	0	0	0	0	0	0.126	0	0.533	0.574
BMP4	TBX5	0	0	0	0	0.054	0.046	0	0.616	0.623
BMP4	KLF4	0	0	0	0	0.042	0.054	0	0.64	0.645
BMP4	SMAD3	0	0	0	0	0.049	0.161	0	0.612	0.663
BMP4	IL6	0	0	0	0	0.055	0	0	0.69	0.694
BMP4	GATA4	0	0	0	0	0.062	0.07	0.75	0.696	0.925
BMP4	BMP2	0	0	0.055	0.96	0.072	0	0.9	0.708	0.97
CACNA1G	SCN5A	0	0	0	0.653	0.082	0.189	0.54	0.269	0.716
CACNA1G	RYR2	0	0	0	0	0.109	0	0	0.567	0.598
CHRD	SMAD3	0	0	0	0	0.055	0.184	0	0.372	0.474
CHRD	GDF11	0	0	0	0	0	0.172	0	0.4	0.482
CHRD	ACVR2A	0	0	0	0	0	0.047	0	0.49	0.493
CHRD	BMP4	0	0	0	0	0	0.474	0.9	0.991	0.999
CHRD	BMP2	0	0	0	0	0	0.474	0.9	0.985	0.999
CNTN2	TNF	0	0	0	0	0	0	0	0.479	0.479
COL1A1	TNFRSF1A	0	0	0	0	0.061	0.046	0	0.467	0.481
COL1A1	KLF4	0	0	0	0	0.044	0.053	0	0.4	0.409
COL1A1	NFKB1	0	0	0	0	0	0.095	0	0.389	0.423
COL1A1	INHBA	0	0	0	0	0.127	0.067	0	0.375	0.446

COL1A1	IL4	0	0	0	0	0	0	0	0.555	0.555
COL1A1	BMP4	0	0	0	0	0.083	0.099	0	0.532	0.579
COL1A1	MEF2C	0	0	0	0	0	0	0	0.584	0.584
COL1A1	IL1B	0	0	0	0	0	0	0	0.596	0.596
COL1A1	TNF	0	0	0	0	0	0.078	0	0.582	0.598
COL1A1	IL6	0	0	0	0	0.063	0	0	0.621	0.63
COL1A1	BMP2	0	0	0	0	0.068	0.099	0	0.662	0.691
COL1A1	SMAD3	0	0	0	0	0	0.126	0	0.751	0.773
COL1A1	COL2A1	0	0	0.054	0.971	0.042	0.075	0.72	0.521	0.867
COL1A1	COL3A1	0	0	0.065	0.95	0.958	0	0.9	0.965	0.999
COL2A1	TNFRSF1A	0	0	0	0	0	0.046	0	0.414	0.417
COL2A1	COL1A1	0	0	0.054	0.971	0.042	0.075	0.72	0.521	0.867
COL2A1	BMP4	0	0	0	0	0.042	0.099	0	0.5	0.531
COL2A1	IL1B	0	0	0	0	0	0	0	0.596	0.596
COL2A1	COL3A1	0	0	0.064	0.951	0.044	0	0.72	0.524	0.864
COL2A1	SMAD3	0	0	0	0	0	0.063	0	0.405	0.418
COL2A1	BMP2	0	0	0	0	0	0.334	0	0.792	0.855
COL2A1	TNF	0	0	0	0	0	0.078	0	0.441	0.463
COL2A1	IL6	0	0	0	0	0	0	0	0.487	0.487
COL3A1	TNFRSF1A	0	0	0	0	0.053	0.046	0	0.501	0.509
COL3A1	BCL3	0	0	0	0	0.056	0.095	0	0.383	0.427
COL3A1	COL1A1	0	0	0.065	0.95	0.958	0	0.9	0.965	0.999
COL3A1	IL4	0	0	0	0	0	0	0	0.428	0.428
COL3A1	INHBA	0	0	0	0	0.101	0.067	0	0.545	0.585
COL3A1	IL1B	0	0	0	0	0	0	0	0.467	0.467
COL3A1	TNF	0	0	0	0	0.042	0.096	0	0.42	0.454
COL3A1	IL6	0	0	0	0	0.06	0	0	0.491	0.501
COL3A1	SMAD3	0	0	0	0	0	0.071	0	0.588	0.601
COL3A1	COL2A1	0	0	0.064	0.951	0.044	0	0.72	0.524	0.864
CSNK1A1	TNFRSF1A	0	0	0	0	0.042	0.292	0	0.435	0.583
CSNK1A1	NFKBIA	0	0	0	0	0.056	0.292	0	0.183	0.406
CSNK1A1	APC	0	0	0	0	0.05	0.292	0.8	0.994	0.999

CSNK1A1	RIPK1	0	0	0	0	0.064	0.292	0	0.463	0.613
CSNK1A1	GSK3B	0	0	0	0	0.067	0.077	0.8	0.996	0.999
DSG2	PKP2	0	0	0	0	0.223	0.36	0.5	0.998	0.999
DSG2	RYR2	0	0	0	0	0	0	0	0.764	0.764
DSG2	SCN5A	0	0	0	0	0	0.045	0	0.767	0.768
ETV1	KLF6	0	0	0	0	0	0.045	0	0.419	0.421
GATA4	BMP4	0	0	0	0	0.062	0.07	0.75	0.696	0.925
GATA4	KLF2	0	0	0	0	0	0.091	0	0.387	0.419
GATA4	KLF15	0	0	0	0	0.044	0.091	0	0.607	0.629
GATA4	KLF13	0	0	0	0	0	0.328	0	0.516	0.661
GATA4	TBX5	0	0	0	0	0.077	0.311	0	0.998	0.998
GATA4	TNNI3K	0	0	0	0	0	0.093	0	0.528	0.553
GATA4	GSK3B	0	0	0	0	0	0.105	0	0.473	0.509
GATA4	SCN5A	0	0	0	0	0.057	0	0	0.479	0.487
GATA4	IRX3	0	0	0	0	0.055	0.068	0	0.398	0.424
GATA4	SMAD3	0	0	0	0	0.055	0.095	0	0.595	0.624
GATA4	MEF2C	0	0	0	0	0.055	0.071	0	0.967	0.969
GATA4	RYR2	0	0	0	0	0.042	0	0	0.506	0.506
GATA4	KLF4	0	0	0	0	0	0.091	0	0.633	0.652
GATA4	KLF5	0	0	0	0	0.042	0.091	0	0.412	0.443
GATA4	BMP2	0	0	0	0	0.042	0.07	0	0.474	0.49
GDF11	CHRD	0	0	0	0	0	0.172	0	0.4	0.482
GDF11	ACVR2A	0	0	0	0	0.048	0.126	0.9	0.994	0.999
GDF11	SMAD3	0	0	0	0	0	0.095	0	0.549	0.575
GDF11	IL6	0	0	0	0	0.055	0	0	0.581	0.587
GSK3B	BCL3	0	0	0	0	0.042	0.331	0	0.528	0.671
GSK3B	NFKBIA	0	0	0	0	0.056	0.095	0	0.545	0.578
GSK3B	RELB	0	0	0	0	0	0.334	0	0.29	0.506
GSK3B	NFKB1	0	0	0	0	0.058	0.331	0	0.582	0.713
GSK3B	BMP4	0	0	0	0	0	0.126	0	0.533	0.574
GSK3B	TRAF2	0	0	0	0	0.068	0.572	0	0.226	0.664
GSK3B	KLF2	0	0	0	0	0	0.315	0	0.282	0.487

GSK3B	APC	0	0	0	0	0.095	0.863	0.9	0.996	0.999
GSK3B	CSNK1A1	0	0	0	0	0.067	0.077	0.8	0.996	0.999
GSK3B	IL1B	0	0	0	0	0.042	0	0	0.636	0.636
GSK3B	GATA4	0	0	0	0	0	0.105	0	0.473	0.509
GSK3B	BMP2	0	0	0	0	0	0.126	0	0.465	0.512
GSK3B	KLF4	0	0	0	0	0	0.073	0	0.512	0.528
GSK3B	TNF	0	0	0	0	0	0.057	0	0.643	0.649
GSK3B	KLF5	0	0	0	0	0	0.526	0	0.314	0.661
GSK3B	IL6	0	0	0	0	0	0	0	0.682	0.682
GSK3B	SMAD3	0	0	0	0	0.074	0.535	0	0.894	0.95
IL1B	TNFRSF1A	0	0	0	0	0.056	0	0	0.997	0.997
IL1B	BCL3	0	0	0	0	0.213	0	0	0.657	0.719
IL1B	RIPK3	0	0	0	0	0.15	0.095	0	0.594	0.66
IL1B	NFKBIA	0	0	0	0	0.283	0	0	0.927	0.945
IL1B	RELB	0	0	0	0	0.154	0	0	0.65	0.691
IL1B	COL1A1	0	0	0	0	0	0	0	0.596	0.596
IL1B	NFKB1	0	0	0	0	0.2	0	0.9	0.959	0.996
IL1B	IL2	0	0	0	0	0.073	0	0	0.999	0.999
IL1B	IL4	0	0	0	0	0.072	0	0.4	0.998	0.998
IL1B	IL5	0	0	0	0	0.104	0	0	0.998	0.998
IL1B	BMP4	0	0	0	0	0.047	0	0	0.457	0.461
IL1B	TRAF2	0	0	0	0	0.067	0.07	0	0.449	0.48
IL1B	KLF2	0	0	0	0	0.076	0	0	0.395	0.417
IL1B	RIPK1	0	0	0	0	0.061	0.095	0	0.606	0.636
IL1B	KLF4	0	0	0	0	0.072	0	0	0.447	0.464
IL1B	COL3A1	0	0	0	0	0	0	0	0.467	0.467
IL1B	COL2A1	0	0	0	0	0	0	0	0.596	0.596
IL1B	BMP2	0	0	0	0	0.08	0	0	0.588	0.605
IL1B	GSK3B	0	0	0	0	0.042	0	0	0.636	0.636
IL1B	SMAD3	0	0	0	0	0.088	0	0	0.618	0.637
IL1B	IL6	0	0	0	0	0.515	0	0.4	0.989	0.996
IL1B	TNF	0	0	0	0	0.616	0	0.4	0.993	0.998

IL2	TNFRSF1A	0	0	0	0	0	0	0	0.74	0.74
IL2	IL2RB	0	0	0	0	0.047	0.979	0.9	0.997	0.999
IL2	NFKBIA	0	0	0	0	0.06	0.046	0	0.574	0.585
IL2	RELB	0	0	0	0	0.047	0.053	0	0.437	0.447
IL2	NFKB1	0	0	0	0	0.055	0.046	0	0.8	0.804
IL2	SMAD3	0	0	0	0	0	0	0	0.472	0.472
IL2	TNF	0	0	0	0	0.072	0	0	0.955	0.957
IL2	IL4	0	0	0	0	0.432	0	0	0.999	0.999
IL2	IL6	0	0	0	0	0.055	0	0	0.999	0.999
IL2	IL1B	0	0	0	0	0.073	0	0	0.999	0.999
IL2	IL5	0	0	0	0	0.266	0	0.4	0.999	0.999
IL2RB	TNF	0	0	0	0	0.122	0	0	0.497	0.54
IL2RB	IL5	0	0	0	0	0	0	0.65	0.377	0.772
IL2RB	IL6	0	0	0	0	0.056	0	0.65	0.608	0.859
IL2RB	IL4	0	0	0	0	0.06	0	0.65	0.767	0.916
IL2RB	IL2	0	0	0	0	0.047	0.979	0.9	0.997	0.999
IL4	TNFRSF1A	0	0	0	0	0	0	0	0.582	0.582
IL4	IL2RB	0	0	0	0	0.06	0	0.65	0.767	0.916
IL4	NFKBIA	0	0	0	0	0.045	0	0	0.587	0.588
IL4	RELB	0	0	0	0	0	0.045	0	0.43	0.432
IL4	COL1A1	0	0	0	0	0	0	0	0.555	0.555
IL4	NFKB1	0	0	0	0	0.045	0	0	0.8	0.8
IL4	IL2	0	0	0	0	0.432	0	0	0.999	0.999
IL4	COL3A1	0	0	0	0	0	0	0	0.428	0.428
IL4	SMAD3	0	0	0	0	0	0	0	0.479	0.479
IL4	TNF	0	0	0	0	0.055	0	0.4	0.963	0.977
IL4	IL6	0	0	0	0	0.044	0	0.4	0.978	0.986
IL4	IL5	0	0	0	0	0.351	0	0	0.983	0.988
IL4	IL1B	0	0	0	0	0.072	0	0.4	0.998	0.998
IL5	TNFRSF1A	0	0	0	0	0	0	0	0.446	0.446
IL5	IL2RB	0	0	0	0	0	0	0.65	0.377	0.772
IL5	NFKBIA	0	0	0	0	0.046	0	0	0.402	0.405

IL5	NFKB1	0	0	0	0	0.046	0	0	0.525	0.527
IL5	IL2	0	0	0	0	0.266	0	0.4	0.999	0.999
IL5	IL4	0	0	0	0	0.351	0	0	0.983	0.988
IL5	TNF	0	0	0	0	0.054	0	0	0.929	0.93
IL5	IL6	0	0	0	0	0.042	0	0	0.977	0.977
IL5	IL1B	0	0	0	0	0.104	0	0	0.998	0.998
IL6	TNFRSF1A	0	0	0	0	0.047	0	0	0.997	0.997
IL6	BCL3	0	0	0	0	0.176	0	0	0.721	0.761
IL6	IL2RB	0	0	0	0	0.056	0	0.65	0.608	0.859
IL6	RIPK3	0	0	0	0	0.054	0	0	0.448	0.455
IL6	NFKBIA	0	0	0	0	0.175	0	0	0.915	0.927
IL6	RELB	0	0	0	0	0.188	0	0	0.629	0.686
IL6	COL1A1	0	0	0	0	0.063	0	0	0.621	0.63
IL6	NFKB1	0	0	0	0	0.176	0	0.9	0.942	0.994
IL6	IL2	0	0	0	0	0.055	0	0	0.999	0.999
IL6	IL4	0	0	0	0	0.044	0	0.4	0.978	0.986
IL6	IL5	0	0	0	0	0.042	0	0	0.977	0.977
IL6	INHBA	0	0	0	0	0.165	0	0	0.311	0.4
IL6	BMP4	0	0	0	0	0.055	0	0	0.69	0.694
IL6	TRAF2	0	0	0	0	0.06	0	0	0.39	0.403
IL6	KLF2	0	0	0	0	0.081	0	0	0.405	0.43
IL6	GDF11	0	0	0	0	0.055	0	0	0.581	0.587
IL6	RIPK1	0	0	0	0	0.083	0	0	0.507	0.529
IL6	IL1B	0	0	0	0	0.515	0	0.4	0.989	0.996
IL6	COL3A1	0	0	0	0	0.06	0	0	0.491	0.501
IL6	GSK3B	0	0	0	0	0	0	0	0.682	0.682
IL6	SMAD3	0	0	0	0	0.084	0	0	0.648	0.664
IL6	KLF4	0	0	0	0	0.1	0	0	0.61	0.634
IL6	BMP2	0	0	0	0	0.091	0	0	0.576	0.599
IL6	COL2A1	0	0	0	0	0	0	0	0.487	0.487
IL6	KLF6	0	0	0	0	0.087	0	0	0.538	0.56
IL6	TNF	0	0	0	0	0.261	0	0.4	0.989	0.994

INHBA	COL1A1	0	0	0	0	0.127	0.067	0	0.375	0.446
INHBA	ACVR2A	0	0	0	0	0.048	0.982	0.9	0.979	0.999
INHBA	IL6	0	0	0	0	0.165	0	0	0.311	0.4
INHBA	COL3A1	0	0	0	0	0.101	0.067	0	0.545	0.585
INHBA	SMAD3	0	0	0	0	0	0.095	0.5	0.637	0.821
IRX3	TBX5	0	0	0	0	0.061	0.046	0	0.488	0.501
IRX3	GATA4	0	0	0	0	0.055	0.068	0	0.398	0.424
KLF10	RELB	0	0	0	0	0.062	0.095	0	0.416	0.461
KLF12	KLF8	0	0	0.056	0.904	0.047	0	0	0.406	0.419
KLF13	GATA4	0	0	0	0	0	0.328	0	0.516	0.661
KLF15	GATA4	0	0	0	0	0.044	0.091	0	0.607	0.629
KLF2	NFKB1	0	0	0	0	0.068	0.091	0.9	0.356	0.938
KLF2	BMP4	0	0	0	0	0	0.054	0	0.445	0.453
KLF2	IL1B	0	0	0	0	0.076	0	0	0.395	0.417
KLF2	GATA4	0	0	0	0	0	0.091	0	0.387	0.419
KLF2	IL6	0	0	0	0	0.081	0	0	0.405	0.43
KLF2	GSK3B	0	0	0	0	0	0.315	0	0.282	0.487
KLF2	TNF	0	0	0	0	0.115	0.058	0	0.446	0.498
KLF2	KLF4	0	0	0	0.857	0.243	0	0	0.463	0.576
KLF4	BCL3	0	0	0	0	0.154	0.091	0	0.465	0.552
KLF4	COL1A1	0	0	0	0	0.044	0.053	0	0.4	0.409
KLF4	NFKB1	0	0	0	0	0.062	0.091	0	0.434	0.475
KLF4	BMP4	0	0	0	0	0.042	0.054	0	0.64	0.645
KLF4	KLF2	0	0	0	0.857	0.243	0	0	0.463	0.576
KLF4	IL1B	0	0	0	0	0.072	0	0	0.447	0.464
KLF4	TBX5	0	0	0	0	0	0.054	0	0.487	0.494
KLF4	GSK3B	0	0	0	0	0	0.073	0	0.512	0.528
KLF4	SMAD3	0	0	0	0	0.067	0.094	0	0.439	0.484
KLF4	MEF2C	0	0	0	0	0.068	0.078	0	0.483	0.518
KLF4	BMP2	0	0	0	0	0.065	0.054	0	0.386	0.409
KLF4	TNF	0	0	0	0	0.106	0.058	0	0.471	0.516
KLF4	IL6	0	0	0	0	0.1	0	0	0.61	0.634

KLF4	GATA4	0	0	0	0	0	0.091	0	0.633	0.652
KLF4	KLF6	0	0	0	0.813	0.236	0.292	0	0.473	0.69
KLF5	NFKBIA	0	0	0	0	0.049	0.091	0	0.452	0.484
KLF5	NFKB1	0	0	0	0	0.062	0.535	0	0.567	0.795
KLF5	GSK3B	0	0	0	0	0	0.526	0	0.314	0.661
KLF5	SMAD3	0	0	0	0	0.067	0.314	0	0.366	0.559
KLF5	GATA4	0	0	0	0	0.042	0.091	0	0.412	0.443
KLF6	BCL3	0	0	0	0	0.081	0.091	0	0.441	0.492
KLF6	NFKBIA	0	0	0	0	0.228	0.328	0	0.234	0.568
KLF6	RELB	0	0	0	0	0.073	0.095	0	0.378	0.432
KLF6	SMAD3	0	0	0	0	0.054	0.071	0	0.563	0.582
KLF6	KLF4	0	0	0	0.813	0.236	0.292	0	0.473	0.69
KLF6	KLF9	0	0	0	0.783	0.076	0	0	0.5	0.519
KLF6	IL6	0	0	0	0	0.087	0	0	0.538	0.56
KLF6	ETV1	0	0	0	0	0	0.045	0	0.419	0.421
KLF8	KLF12	0	0	0.056	0.904	0.047	0	0	0.406	0.419
KLF9	KLF6	0	0	0	0.783	0.076	0	0	0.5	0.519
MEF2C	COL1A1	0	0	0	0	0	0	0	0.584	0.584
MEF2C	BMP4	0	0	0	0	0	0	0	0.529	0.529
MEF2C	TBX5	0	0	0	0	0	0.053	0	0.913	0.914
MEF2C	SMAD3	0	0	0	0	0.048	0.046	0	0.689	0.693
MEF2C	RYR2	0	0	0	0	0.055	0	0	0.43	0.438
MEF2C	KLF4	0	0	0	0	0.068	0.078	0	0.483	0.518
MEF2C	GATA4	0	0	0	0	0.055	0.071	0	0.967	0.969
NFKB1	TNFRSF1A	0	0	0	0	0.063	0	0	0.844	0.848
NFKB1	BCL3	0	0	0	0.662	0.175	0.835	0.9	0.984	0.999
NFKB1	RIPK3	0	0	0	0	0.073	0.095	0.5	0.613	0.815
NFKB1	NFKBIA	0	0	0	0.755	0.242	0.997	0.9	0.986	0.999
NFKB1	RELB	0	0	0	0.743	0.323	0.93	0.54	0.986	0.999
NFKB1	COL1A1	0	0	0	0	0	0.095	0	0.389	0.423
NFKB1	KLF4	0	0	0	0	0.062	0.091	0	0.434	0.475
NFKB1	IL5	0	0	0	0	0.046	0	0	0.525	0.527

NFKB1	GSK3B	0	0	0	0	0.058	0.331	0	0.582	0.713
NFKB1	TRAF2	0	0	0	0	0.083	0.045	0.5	0.433	0.718
NFKB1	SMAD3	0	0	0	0	0.135	0.046	0	0.738	0.765
NFKB1	KLF5	0	0	0	0	0.062	0.535	0	0.567	0.795
NFKB1	IL4	0	0	0	0	0.045	0	0	0.8	0.8
NFKB1	IL2	0	0	0	0	0.055	0.046	0	0.8	0.804
NFKB1	RIPK1	0	0	0	0	0.192	0.095	0.5	0.748	0.895
NFKB1	KLF2	0	0	0	0	0.068	0.091	0.9	0.356	0.938
NFKB1	IL6	0	0	0	0	0.176	0	0.9	0.942	0.994
NFKB1	IL1B	0	0	0	0	0.2	0	0.9	0.959	0.996
NFKB1	TNF	0	0	0	0	0.194	0	0.9	0.995	0.999
NFKBIA	TNFRSF1A	0	0	0	0	0.057	0	0	0.806	0.809
NFKBIA	BCL3	0	0	0.186	0.681	0.257	0	0.72	0.5	0.904
NFKBIA	RIPK3	0	0	0	0	0.067	0.095	0.5	0.41	0.717
NFKBIA	IL5	0	0	0	0	0.046	0	0	0.402	0.405
NFKBIA	CSNK1A1	0	0	0	0	0.056	0.292	0	0.183	0.406
NFKBIA	SMAD3	0	0	0	0	0.044	0.046	0	0.469	0.473
NFKBIA	KLF5	0	0	0	0	0.049	0.091	0	0.452	0.484
NFKBIA	KLF6	0	0	0	0	0.228	0.328	0	0.234	0.568
NFKBIA	GSK3B	0	0	0	0	0.056	0.095	0	0.545	0.578
NFKBIA	IL2	0	0	0	0	0.06	0.046	0	0.574	0.585
NFKBIA	IL4	0	0	0	0	0.045	0	0	0.587	0.588
NFKBIA	TRAF2	0	0	0	0	0.063	0.045	0.5	0.529	0.761
NFKBIA	RIPK1	0	0	0	0	0.05	0.095	0.5	0.599	0.804
NFKBIA	IL6	0	0	0	0	0.175	0	0	0.915	0.927
NFKBIA	IL1B	0	0	0	0	0.283	0	0	0.927	0.945
NFKBIA	TNF	0	0	0	0	0.338	0.292	0	0.937	0.967
NFKBIA	RELB	0	0.001	0	0	0.181	0.861	0	0.963	0.995
NFKBIA	NFKB1	0	0	0	0.755	0.242	0.997	0.9	0.986	0.999
PKP2	RYR2	0	0	0	0	0	0	0	0.828	0.828
PKP2	SCN5A	0	0	0	0	0	0	0	0.976	0.976
PKP2	DSG2	0	0	0	0	0.223	0.36	0.5	0.998	0.999

RELB	TNFRSF1A	0	0	0	0	0.092	0	0	0.594	0.615
RELB	BCL3	0	0.105	0	0	0.475	0.344	0	0.882	0.959
RELB	NFKBIA	0	0.001	0	0	0.181	0.861	0	0.963	0.995
RELB	IL4	0	0	0	0	0	0.045	0	0.43	0.432
RELB	KLF6	0	0	0	0	0.073	0.095	0	0.378	0.432
RELB	IL2	0	0	0	0	0.047	0.053	0	0.437	0.447
RELB	KLF10	0	0	0	0	0.062	0.095	0	0.416	0.461
RELB	TRAF2	0	0	0	0	0.117	0.045	0	0.415	0.464
RELB	GSK3B	0	0	0	0	0	0.334	0	0.29	0.506
RELB	SMAD3	0	0	0	0	0.103	0.293	0	0.286	0.508
RELB	RIPK1	0	0	0	0	0.137	0.098	0	0.436	0.523
RELB	IL6	0	0	0	0	0.188	0	0	0.629	0.686
RELB	IL1B	0	0	0	0	0.154	0	0	0.65	0.691
RELB	TNF	0	0	0	0	0.195	0.048	0	0.699	0.749
RELB	NFKB1	0	0	0	0.743	0.323	0.93	0.54	0.986	0.999
RIPK1	TNFRSF1A	0	0	0	0	0.111	0.994	0.9	0.997	0.999
RIPK1	RIPK3	0	0	0.135	0.663	0.062	0.999	0.9	0.988	0.999
RIPK1	NFKBIA	0	0	0	0	0.05	0.095	0.5	0.599	0.804
RIPK1	RELB	0	0	0	0	0.137	0.098	0	0.436	0.523
RIPK1	NFKB1	0	0	0	0	0.192	0.095	0.5	0.748	0.895
RIPK1	TRAF2	0	0	0	0	0.075	0.994	0.9	0.994	0.999
RIPK1	BMP2	0	0	0	0	0.044	0.126	0	0.392	0.447
RIPK1	IL6	0	0	0	0	0.083	0	0	0.507	0.529
RIPK1	CSNK1A1	0	0	0	0	0.064	0.292	0	0.463	0.613
RIPK1	IL1B	0	0	0	0	0.061	0.095	0	0.606	0.636
RIPK1	TNF	0	0	0	0	0.1	0.994	0.5	0.943	0.999
RIPK3	TNFRSF1A	0	0	0	0	0.115	0.311	0	0.891	0.927
RIPK3	IL6	0	0	0	0	0.054	0	0	0.448	0.455
RIPK3	IL1B	0	0	0	0	0.15	0.095	0	0.594	0.66
RIPK3	NFKBIA	0	0	0	0	0.067	0.095	0.5	0.41	0.717
RIPK3	NFKB1	0	0	0	0	0.073	0.095	0.5	0.613	0.815
RIPK3	TRAF2	0	0	0	0	0.055	0.328	0.9	0.498	0.963

RIPK3	TNF	0	0	0	0	0.137	0.994	0	0.76	0.998
RIPK3	RIPK1	0	0	0.135	0.663	0.062	0.999	0.9	0.988	0.999
RYR2	PKP2	0	0	0	0	0	0	0	0.828	0.828
RYR2	DSG2	0	0	0	0	0	0	0	0.764	0.764
RYR2	TBX5	0	0	0	0	0.045	0	0	0.57	0.571
RYR2	SCN5A	0	0	0	0	0.108	0.085	0	0.872	0.887
RYR2	MEF2C	0	0	0	0	0.055	0	0	0.43	0.438
RYR2	ATP2A1	0	0	0	0	0.11	0.103	0	0.527	0.59
RYR2	CACNA1G	0	0	0	0	0.109	0	0	0.567	0.598
RYR2	GATA4	0	0	0	0	0.042	0	0	0.506	0.506
SCN5A	PKP2	0	0	0	0	0	0	0	0.976	0.976
SCN5A	DSG2	0	0	0	0	0	0.045	0	0.767	0.768
SCN5A	TBX5	0	0	0	0	0.067	0.045	0	0.768	0.775
SCN5A	GATA4	0	0	0	0	0.057	0	0	0.479	0.487
SCN5A	CACNA1G	0	0	0	0.653	0.082	0.189	0.54	0.269	0.716
SCN5A	RYR2	0	0	0	0	0.108	0.085	0	0.872	0.887
SMAD3	CHRD	0	0	0	0	0.055	0.184	0	0.372	0.474
SMAD3	NFKBIA	0	0	0	0	0.044	0.046	0	0.469	0.473
SMAD3	RELB	0	0	0	0	0.103	0.293	0	0.286	0.508
SMAD3	COL1A1	0	0	0	0	0	0.126	0	0.751	0.773
SMAD3	NFKB1	0	0	0	0	0.135	0.046	0	0.738	0.765
SMAD3	IL2	0	0	0	0	0	0	0	0.472	0.472
SMAD3	IL4	0	0	0	0	0	0	0	0.479	0.479
SMAD3	ACVR2A	0	0	0	0	0.086	0.126	0.9	0.768	0.979
SMAD3	INHBA	0	0	0	0	0	0.095	0.5	0.637	0.821
SMAD3	BMP4	0	0	0	0	0.049	0.161	0	0.612	0.663
SMAD3	GDF11	0	0	0	0	0	0.095	0	0.549	0.575
SMAD3	IL1B	0	0	0	0	0.088	0	0	0.618	0.637
SMAD3	COL3A1	0	0	0	0	0	0.071	0	0.588	0.601
SMAD3	GSK3B	0	0	0	0	0.074	0.535	0	0.894	0.95
SMAD3	COL2A1	0	0	0	0	0	0.063	0	0.405	0.418
SMAD3	KLF4	0	0	0	0	0.067	0.094	0	0.439	0.484

SMAD3	KLF5	0	0	0	0	0.067	0.314	0	0.366	0.559
SMAD3	KLF6	0	0	0	0	0.054	0.071	0	0.563	0.582
SMAD3	GATA4	0	0	0	0	0.055	0.095	0	0.595	0.624
SMAD3	TNF	0	0	0	0	0.086	0	0	0.613	0.632
SMAD3	BMP2	0	0	0	0	0	0.161	0	0.587	0.638
SMAD3	IL6	0	0	0	0	0.084	0	0	0.648	0.664
SMAD3	MEF2C	0	0	0	0	0.048	0.046	0	0.689	0.693
TBX5	BMP4	0	0	0	0	0.054	0.046	0	0.616	0.623
TBX5	KLF4	0	0	0	0	0	0.054	0	0.487	0.494
TBX5	IRX3	0	0	0	0	0.061	0.046	0	0.488	0.501
TBX5	RYR2	0	0	0	0	0.045	0	0	0.57	0.571
TBX5	SCN5A	0	0	0	0	0.067	0.045	0	0.768	0.775
TBX5	MEF2C	0	0	0	0	0	0.053	0	0.913	0.914
TBX5	GATA4	0	0	0	0	0.077	0.311	0	0.998	0.998
TNF	TNFRSF1A	0	0	0	0	0.044	0.999	0.9	0.999	0.999
TNF	BCL3	0	0	0	0	0.163	0	0	0.473	0.54
TNF	IL2RB	0	0	0	0	0.122	0	0	0.497	0.54
TNF	RIPK3	0	0	0	0	0.137	0.994	0	0.76	0.998
TNF	NFKBIA	0	0	0	0	0.338	0.292	0	0.937	0.967
TNF	RELB	0	0	0	0	0.195	0.048	0	0.699	0.749
TNF	COL1A1	0	0	0	0	0	0.078	0	0.582	0.598
TNF	NFKB1	0	0	0	0	0.194	0	0.9	0.995	0.999
TNF	IL2	0	0	0	0	0.072	0	0	0.955	0.957
TNF	IL4	0	0	0	0	0.055	0	0.4	0.963	0.977
TNF	IL5	0	0	0	0	0.054	0	0	0.929	0.93
TNF	BMP4	0	0	0	0	0.056	0	0	0.444	0.452
TNF	TRAF2	0	0	0	0	0.069	0.994	0.5	0.917	0.999
TNF	KLF2	0	0	0	0	0.115	0.058	0	0.446	0.498
TNF	RIPK1	0	0	0	0	0.1	0.994	0.5	0.943	0.999
TNF	IL1B	0	0	0	0	0.616	0	0.4	0.993	0.998
TNF	COL3A1	0	0	0	0	0.042	0.096	0	0.42	0.454
TNF	GSK3B	0	0	0	0	0	0.057	0	0.643	0.649

TNF	CNTN2	0	0	0	0	0	0	0	0.479	0.479
TNF	SMAD3	0	0	0	0	0.086	0	0	0.613	0.632
TNF	KLF4	0	0	0	0	0.106	0.058	0	0.471	0.516
TNF	BMP2	0	0	0	0	0.058	0	0	0.726	0.731
TNF	COL2A1	0	0	0	0	0	0.078	0	0.441	0.463
TNF	IL6	0	0	0	0	0.261	0	0.4	0.989	0.994
TNFRSF1A	COL2A1	0	0	0	0	0	0.046	0	0.414	0.417
TNFRSF1A	IL5	0	0	0	0	0	0	0	0.446	0.446
TNFRSF1A	COL1A1	0	0	0	0	0.061	0.046	0	0.467	0.481
TNFRSF1A	COL3A1	0	0	0	0	0.053	0.046	0	0.501	0.509
TNFRSF1A	BCL3	0	0	0	0	0.276	0	0	0.351	0.51
TNFRSF1A	IL4	0	0	0	0	0	0	0	0.582	0.582
TNFRSF1A	CSNK1A1	0	0	0	0	0.042	0.292	0	0.435	0.583
TNFRSF1A	RELB	0	0	0	0	0.092	0	0	0.594	0.615
TNFRSF1A	IL2	0	0	0	0	0	0	0	0.74	0.74
TNFRSF1A	NFKBIA	0	0	0	0	0.057	0	0	0.806	0.809
TNFRSF1A	NFKB1	0	0	0	0	0.063	0	0	0.844	0.848
TNFRSF1A	RIPK3	0	0	0	0	0.115	0.311	0	0.891	0.927
TNFRSF1A	IL6	0	0	0	0	0.047	0	0	0.997	0.997
TNFRSF1A	IL1B	0	0	0	0	0.056	0	0	0.997	0.997
TNFRSF1A	TNF	0	0	0	0	0.044	0.999	0.9	0.999	0.999
TNFRSF1A	RIPK1	0	0	0	0	0.111	0.994	0.9	0.997	0.999
TNFRSF1A	TRAF2	0	0	0	0	0	0.994	0.9	0.992	0.999
TNNI3K	GATA4	0	0	0	0	0	0.093	0	0.528	0.553
TRAF2	TNFRSF1A	0	0	0	0	0	0.994	0.9	0.992	0.999
TRAF2	RIPK3	0	0	0	0	0.055	0.328	0.9	0.498	0.963
TRAF2	NFKBIA	0	0	0	0	0.063	0.045	0.5	0.529	0.761
TRAF2	RELB	0	0	0	0	0.117	0.045	0	0.415	0.464
TRAF2	NFKB1	0	0	0	0	0.083	0.045	0.5	0.433	0.718
TRAF2	IL6	0	0	0	0	0.06	0	0	0.39	0.403
TRAF2	IL1B	0	0	0	0	0.067	0.07	0	0.449	0.48
TRAF2	GSK3B	0	0	0	0	0.068	0.572	0	0.226	0.664

TRAF2	TNF	0	0	0	0	0.069	0.994	0.5	0.917	0.999
TRAF2	RIPK1	0	0	0	0	0.075	0.994	0.9	0.994	0.999
