# Supplementary figures and images for: Temporal Orchestration of Krüppel-like Factors During Cardiac Remodeling Following Isoproterenol-Induced Myocardial Injury
Source: Genes (Basel). 2026 Jun 3;17(6):657. doi: 10.3390/genes17060657 (PMC13299128; doi:10.3390/genes17060657)

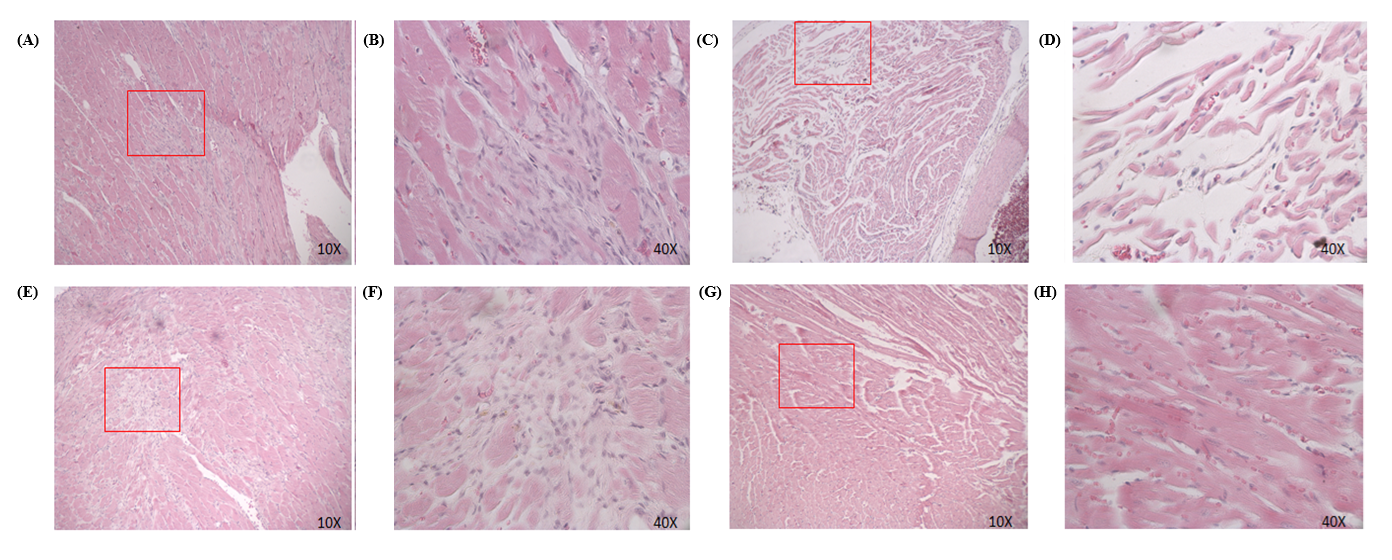

Supplement: Supplementary file 1 [file genes-17-00657-s001.zip › Supplemental Figure S1.png]

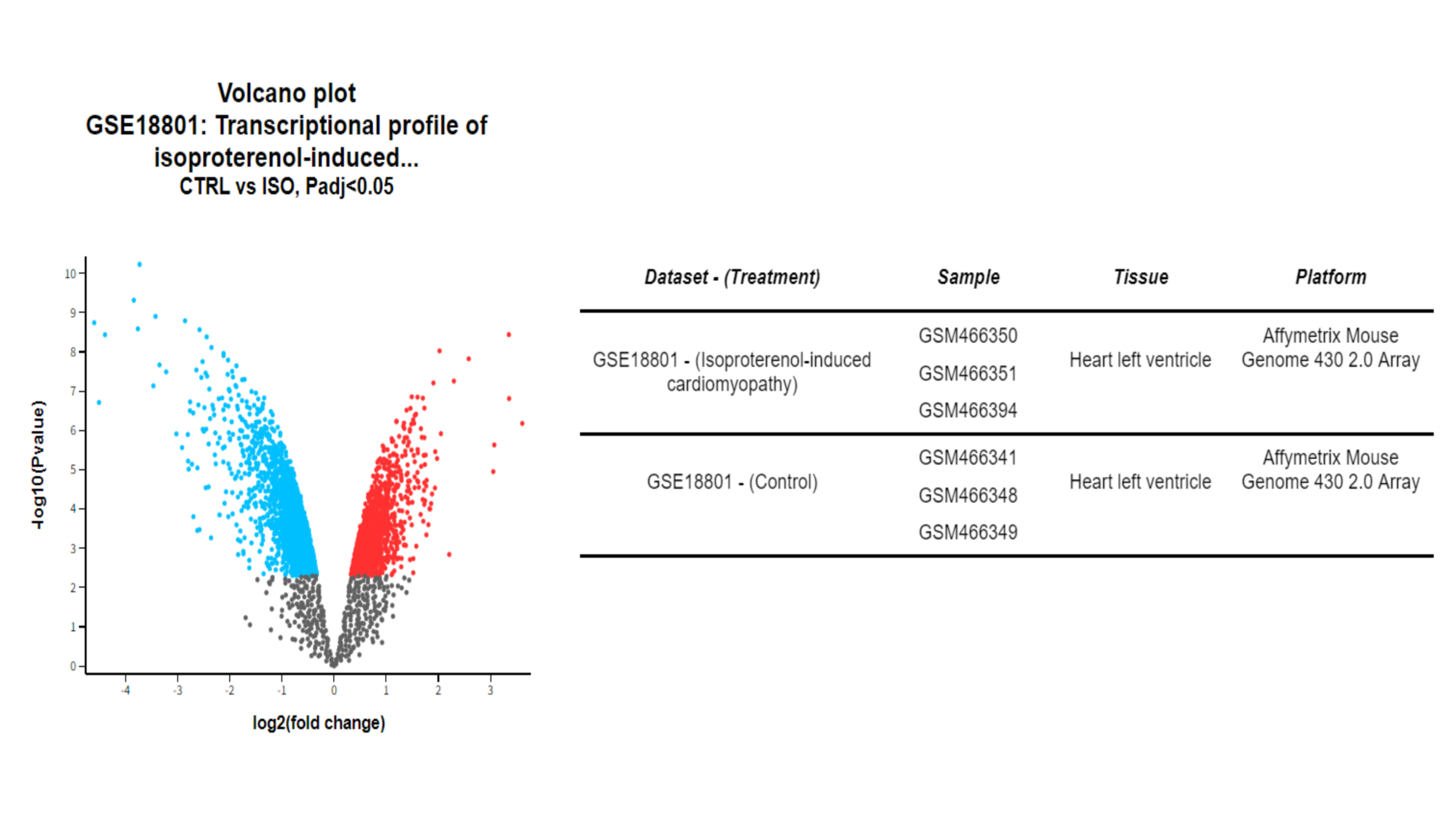

Supplement: Supplementary file 1 [file genes-17-00657-s001.zip › Supplemental Figure S2.TIF]
